# An efficient anoxic/aerobic/aerobic/anoxic process for domestic sewage treatment: From feasibility to application

**DOI:** 10.3389/fmicb.2022.970548

**Published:** 2022-08-02

**Authors:** Yao Wang, Baorui Liang, Fei Kang, Youzhao Wang, Chaoyue Zhao, Zhenning Lyu, Tong Zhu, Zhijun Zhang

**Affiliations:** School of Mechanical Engineering and Automation, Institute of Process Equipment and Environmental Engineering, Northeastern University, Shenyang, China

**Keywords:** biofilm, domestic sewage, elemental-sulfur-based autotrophic denitrification, multi-chamber, no sludge reflux, vibration

## Abstract

In this paper, the anoxic/aerobic/aerobic/anoxic (AOOA) process was proposed using fixed biofilms in a continuous plug-flow multi-chamber reactor, and no sludge reflux operation was performed during the 190 days of operation. The reactor volume ratio of 1.5:2:1.5:1 (A/O/O/A) with the dissolved oxygen (DO) concentration of 2 mg L^−1^ in the aerobic zone was the optimal condition for reactor operation. According to the results obtained from the treatment of real domestic sewage, when the hydraulic retention time (HRT) was 6 h, the effluent of the reactor could meet the discharge standard even in cold conditions (13°C). Specifically, the elemental-sulfur-based autotrophic denitrification (ESAD) process contributed the most to the removal of total inorganic nitrogen (TIN) in the reactor. In addition, the use of vibration method was helpful in removing excess sludge from the biofilms of the reactor. Overall, the AOOA process is an efficient and convenient method for treating domestic sewage.

## Introduction

Nitrogen pollution in water bodies is already a worldwide issue, and excessive accumulation of nutrients is the main cause of this problem (Wang et al., 2019). In China, there are still many water bodies suffering from eutrophication, which is most likely caused by nitrogen pollution (Jiang et al., 2019). Although the Chinese Government agency stipulates that the total nitrogen concentration in the effluent of wastewater treatment plants (WWTPs) cannot be higher than 15 mg L^−1^, due to the low content of organic matter in the influent, many WWTPs find it difficult to meet this discharge standard (Feng et al., 2021). Thus, the development of efficient technology to further improve the effluent quality of WWTPs is urgently needed.

For the WWTPs, nitrification and denitrification (ND) processes are often used due to their high efficiencies. However, when the amount of organic matter in the influent of ND process is not enough to complete denitrification, additional organic matter is needed, which will increase the difficulty and cost of operation. From this point of view, to reduce the dependence on organic matter in sewage treatment processes and the cost, many processes have been proposed, including the partial nitrification–denitrification (PND), anaerobic ammonia oxidation (anammox), partial nitritation-anammox (PN-A), and partial-denitrification-anammox (PD-A; Zhang et al., 2018; Du et al., 2020; Peng et al., 2020; Feng et al., 2021). Nonetheless, the implementation of these processes requires stringent conditions, which limits their widespread application. For instance, the slow growth of anammox bacteria and the need for strict restriction of nitrite-oxidizing bacteria (NOB) limit the application of anammox-based processes for nitrogen removal from sewage (Zhang et al., 2019; Du et al., 2020). Moreover, the ever-changing influent concentration of dissolved oxygen (DO) and ammonium will affect the performance of partial nitrification process, thus resulting in the instability of PND system (Li et al., 2020).

Compared with other reactors, the multi-chamber reactor has shown to be helpful in improving the removal efficiency of pollutants in water bodies (Liang et al., 2021a). Furthermore, the biofilm reactor can help to improve the quality of effluent and reduce the loss of bacteria (Zhang et al., 2015), whereas the multi-chamber reactor coupled with biofilm may improve the capacity of domestic sewage treatment. Nevertheless, the effects of structural changes of the biofilm-coupled multi-chamber reactor on domestic sewage treatment have not been studied in detail. Moreover, as an effective method for removing pollutants from water bodies, the effect of vibration method on the treatment of domestic sewage after coupling biofilms with multi-chamber reactor is unclear.

In this study, a lab-scale multi-chamber reactor coupled with biofilm was tested under different conditions for the treatment of domestic sewage. The objectives of this study were: (1) to compare the influence of changes in the structure of the reactor on the treatment of domestic wastewater; (2) to study the performance of the reactor under different DO concentrations and influent chemical oxygen demand/total inorganic nitrogen (COD/TIN) ratios; (3) to study the impact of vibration method on the reactor; (4) to analyze the changes in the microbial community in the reactor at different times. The results of this study would help to simplify the operation of domestic sewage treatment and improve the treatment capacity.

## Materials and methods

### Reactor configuration

**Figure 1:**
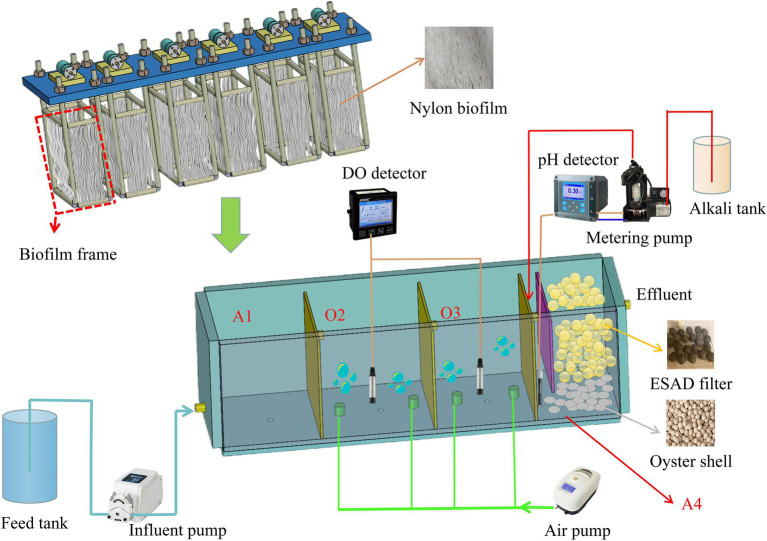
The schematic diagram of the reactor.

A continuous plug-flow reactor with an effective working volume of 15.4 l was used in this study. The reactor was divided into four different chambers by three baffles, and the chambers were orderly adopted as the anoxic part 1 (A1), aerobic part 2 (O2), aerobic part 3 (O3), and anoxic part 4 (A4; Figure 1). The reactor was made of plexiglass with a rectangular shape, had a width of 15 cm, an effective water height of 17 cm, and a length of 60.4 cm. The baffle position between chambers A1 and O2 was made movable so as to allow easy adjustability, yielding the desired working volumes in A1 and O2 at any moment. This was used to study the effect of changes in the volumes of A1 and O2 on the removal of pollutants, and the working volumes of O3 and A4 were fixed at 3.8 and 2.6 l, respectively. The pH probe connected to a pH controller (MIK-pH 8.0, Meacon, China) was used to control the metering pump (DS2-PU2, Xin Xishan, China), which was used to maintain the influent pH of A4 at about 7.0 ± 0.1.

The biofilm frame had a rectangular shape and was welded using several stainless-steel rods, with a width of 13 cm, a height of 22 cm, and a length of 5 cm. Two identical biofilm frames were filled into the A1, O2, and O3 chambers, respectively, and the nylon biofilms were immobilized on biofilm frames to serve as the carriers for microorganisms (Figure 1). The biofilm frames were combined with a rectangular plate, and six eccentric DC motors (BGB37-528, Baishizhili, China, 12 V DC motor) were installed on the top of the plate. The vibration of biofilm frames was driven by operating motors, and a DC speed regulator (DC-12 V, Gewa, China) was adopted to regulate the motor speed.

### Seed sludge and sewage

The synthetic domestic sewage used for the influent was made from tap water supplemented with glucose and NH_4_Cl as the COD and ammonium sources, respectively. The NaHCO_3_ (0.7 g l^−1^) solution was used as the pH regulator for the A4 chamber. The seed sludge of the O2 and O3 chambers was purchased from an environmental technology company in Shenyang (Dongyuan, China), which is applied for the ND process of the domestic sewage. For the A1 and A4 chambers, the seed sludge was obtained from Donggang WWTP (Rizhao, China). The real domestic sewage adopted in the reactor was obtained from the Donggang WWTP, its main compounds are presented in Table 1.

**Table 1:** Influent parameters of the reactor.

Periods	I	II	III	IV
Days	1–70	71–110	111–160	161–190
HRT (h)	12–4	5	5	5 and 6
COD/TIN	4	4	2–6	3.5
Average temperature (°C)		29 ± 2	13 ± 2
Ammonium (mg L^−1^)		50	48.2 ± 15.3
Sulfate (mg L^−1^)		52.3 ± 4.6	69.3 ± 5.9
COD (mg L^−1^)	200		100–300	168.7 ± 30.6
DO concentration of O2 and O3 (mg L^−1^)	3	1–4	2	

### Reactor start-up and experimental procedure

In the A4 chamber, 800 g of oyster shell was evenly filled into the lower part to further improve the pH value, and 1,800 g of the elemental-sulfur-based autotrophic denitrification (ESAD) filter was evenly filled into the upper part for the denitrification process (Figure 1). The composition and structure of the ESAD filter were consistent with those of the USS filter used in our previous study (Liang et al., 2020). The ESAD process was adopted because of its low sludge yield and good denitrification capacity as compared to other denitrification processes (Sahinkaya et al., 2014). The size of the oyster shell and the ESAD filter were screened at around 6 cm^3^. The volume of the seed sludge [the concentration of mixed liquor suspended solids (MLSS) was around 4.1 g L^−1^] which was inoculated into the A1 and A4 chambers in volumes of 600 and 400 ml, respectively, while for O2 and O3 inoculated sludge (with the MLSS concentration of around 3.8 g L^−1^), these volumes were 800 and 600 ml, respectively. After the reactor was filled with seed sludge, the motors were turned on to promote the vibration of biofilm frames to help the seed sludge attach onto the nylon biofilm carriers. This situation lasted for 24 h before continuous feeding experiments, the DO concentration of O2 and O3 was maintained at around 3 mg L^−1^ until period II.

After inoculation, the reactor was operated for 190 days with continuous feeding and was divided into four periods. Period I was planned to test the reactor performance under different hydraulic retention times (HRT), and to compare the performance of reactor under different volume ratios, the volume ratios of A1/O2 were changed (Table 2). During period II, to investigate the effect of changes in the concentration of DO on the reactor, the DO concentrations of O2 and O3 chambers were changed and two batches of repeated experiments were carried out. Similarly, batch experiments were carried out in period III to study the effects of different COD/TIN ratios on the reactor (Table 3), and throughout periods I to III, the water temperature of the reactor was controlled at 29°C ± 2°C using heaters.

**Table 2:** Operating parameters of the reactor during period I.

Days	1–5	6–9	10–13	14–20	21–25	26–30	31–35 and 51–55	36–40 and 56–60	41–45 and 61–65	46–50 and 66–70
**Operation conditions**										
HRT (h)	12	8	6	5	4	5	5	5	5	5
Volume ratio (A1/O2/O3/A4)	2:1.5:1.5:1						2:1.5:1.5:1	2.5:1:1.5:1	1.5:2:1.5:1	1:2.5:1.5:1
Influent COD (mg L^−1^)					200				
Influent ammonium (mg L^−1^)					50				
**Effluent characteristics**										
COD (mg L^−1^)	10.3 ± 2.3	10.7 ± 2.6	13.1 ± 3.2	12.4 ± 2.3	24.4 ± 8.7	12.8 ± 3.6	18.2 ± 6.8	11.1 ± 4.3	15.7 ± 3.9	23.1 ± 11.6
Ammonium (mg L^−1^)	2.1 ± 1.1	3.5 ± 0.4	4.2 ± 0.6	2.9 ± 0.9	10.6 ± 2.6	3.6 ± 1.5	3.9 ± 0.9	12.3 ± 2.6	1.95 ± 0.4	4.6 ± 0.7
TNE (mg L^−1^)	3.1 ± 1.3	8.2 ± 1.5	10.1 ± 1.6	6.2 ± 1.1	16.8 ± 3.1	7.6 ± 0.8	11.3 ± 1.4	18.3 ± 3.2	3.5 ± 0.8	10.5 ± 1.3
COD removal efficiency (%)	94.9	94.7	93.5	93.7	87.8	93.6	90.9	94.4	92.1	88.4
ARE (%)	95.8	93	91.6	94.2	78.8	92.8	92.2	75.4	96.1	90.8

**Table 3:** Operation parameters of the reactor during periods II and III.

Periods		II				III				
Days	Batch 1	71–75	76–80	81–85	86–90	111–115	116–120	121–125	126–130	131–135
	Batch 2	91–95	96–100	101–105	106–110	136–140	141–145	146–150	151–155	156–160
DO concentration of O2 and O3 (mg L^−1^)		1	2	3	4	2				
Influent COD/TIN		4				2	3	4	5	6
Influent COD (mg L^−1^)		200				100	150	200	250	300

During period IV, to study the effects of real domestic sewage and changes in ambient temperature, the heaters in the reactor were turned off to keep the reactor operating at ambient temperatures, and the influent of the reactor was changed from synthetic sewage to real sewage (Table 1). No reflux operation was performed throughout the study, and during days 120–190, to mitigate the impact of sludge accumulation on the biofilm frames, vibrations were performed every 20 days, which lasted for 1 h each time. According to the actual conditions of the vibrations of the biofilm frames, the speed of the motor was stabilized at 600 r/min during the vibrations. After the vibrations, the reactor was briefly stopped (for 15 min), thus temporal cessation of sewage feed supply, allowing the suspended sludge to settle, and then the sludge was discharged through the outlet at the bottom of the reactor. The HRT was calculated considering the empty bed volume, and the following equations were used to calculate the HRT, TIN, and total nitrogen in effluent (TNE):


(1) $$HRT=\frac{B_{a}}{R_{b}}$$

(2) $$\mathrm{TIN}=N_{a}+N_{i}+N_{N}$$

(3) $$TNE=E_{a}+E_{i}+E_{N}$$

where *R_b_* is the influent flow rate of the reactor (L h^−1^); *B_a_* is the empty bed volume of the reactor (L); *Na*, *Ni*, and *NN* are the ammonium, nitrate, and nitrite concentrations (mg L^−1^) in the influent of the reactor, respectively; *Ea*, *Ei*, and *EN* are the ammonium, nitrate, and nitrite concentrations (mg L^−1^) in the effluent of the reactor, respectively.

### Analytical methods

The samples collected from the inlet, A1, O2, O3, and A4 (outlet) were tested on daily basis. Before the measurements, the 0.45 μm membrane filters were adopted to filter the samples, then the following parameters were analyzed according to APHA (2005): COD, sulfate, MLSS, nitrate, nitrite, and ammonium. For the sulfate, the samples were only collected from the inlet and A4 effluent. The concentration of MLSS in the effluent from A4 chamber was monitored once a week. The digital probes (Pro20i, YSI, United States and PHS-3C, LeiCi, China) were, respectively, adopted to measure the DO and pH values.

### DNA extraction and Illumina MiSeq sequencing

Microbial samples were collected from different chambers of the reactor to study microbial communities. For the A1, O2, and O3 chambers, the sludge was taken from four different points on the two biofilm frames in each chamber and then mixed to form the finished sample, while for the finished sample of A4 chamber, the sludge on the surface of ESAD filters was collected from four different points and mixed. The samples were collected on day 30 and 190, respectively, and one sample was taken at a time from each chamber. The PowerSoil DNA extraction kit (FastDNA Spin Kit for Soil, MP Biomedicals, United States) was used to extract the DNA, and according to the standard protocols (Majorbio, Shanghai, China), the V3–V4 hypervariable region of the bacterial 16S rRNA gene was targeted by primers 338F (5′-ACTCCTACGGGAGGCAGCAG-3′) and 806R (5′-GGACTACHVGGGTWTCTAAT-3′). Both the amplicons were pooled in equimolar, and paired-end sequenced (2 × 300) on a MiSeq sequencing platform (Illumina, San Diego, USA).

## Results and discussion

### Performance of the reactor under different HRTs and volume ratios

During period I, the variations in COD and nitrogen concentrations of the reactor are shown in Table 2. On days 1–30, to study the effect of changes in influent flow rate on the reactor, the overall performance of the reactor was observed under different HRTs. After 5 days of operation, the average effluent COD, ammonium, and TNE concentrations of the reactor were 10.3 ± 2.3, 2.1 ± 1.1, and 3.1 ± 1.3 mg L^−1^, respectively, indicating that the reactor can start up quickly and achieve the desired pollutant removal performance. When the COD and ammonium effluent concentrations were, respectively, lower than 50 and 5 mg L^−1^ for three consecutive days, the HRT of the reactor gradually decreased. Throughout period I, the COD concentration in the effluent of the reactor was always lower than 50 mg L^−1^, while the average ammonium concentration increased to 10.6 ± 2.6 mg L^−1^ when the HRT was 4 h. This may have been caused by the faster flow rate of sewage that weakened the treatment efficiency of ammonium in the reactor. Therefore, the HRT of the reactor was stabilized at 5 h, and then the two repeat batches of volume change experiments were performed at 30–70 days.

The A1/O2/O3/A4 volume ratio was first set at 2:1.5:1.5:1, which was then successively changed to 2.5:1:1.5:1, 1.5:2:1.5:1, and 1:2.5:1.5:1, respectively. The reactor was run continuously for 5 days at each volume ratio, and this was repeated on days 51–70. Under the four different volume ratios, the COD concentrations of the effluent from the reactor were always lower than 50 mg L^−1^, and the removal of COD was mainly carried out in the A1 chamber. Compared to other volume ratios, the reactor obtained the lowest COD removal efficiency when the ratio was 1:2.5:1.5:1, with the effluent concentration of COD was 23.1 ± 11.6 mg L^−1^ (Table 2).

The change of the volume has a great influence on the concentration of nitrogen in the effluent of the reactor, the effluent average ammonium concentration of 12.3 ± 2.6 mg L^−1^ was obtained under the volume ratio of 2.5:1:1.5:1 in period I. This could be attributed to the decrease in the volume of aerobic zone, whereas for the other three volume ratios, due to the larger volumes of the aerobic zone, the ammonium removal efficiencies (ARE) of the reactor were both greater than 90%. The optimal ARE was 96.1% by volume ratio of 1.5:2:1.5:1, with the effluent average TNE concentration of 3.5 ± 0.8 mg L^−1^. In a word, the application of anoxic/aerobic/aerobic/anoxic (AOOA) process in the reactor shows that the reactor can start-up quickly and efficiently treat COD and TIN in domestic sewage, the optimal volume ratio is considered to be 1.5:2:1.5:1 in the experiment, and the volume ratio of the reactor was fixed at 1.5:2:1.5:1 in the next periods.

### Performance of the reactor under different DO concentrations

**Figure 2:**
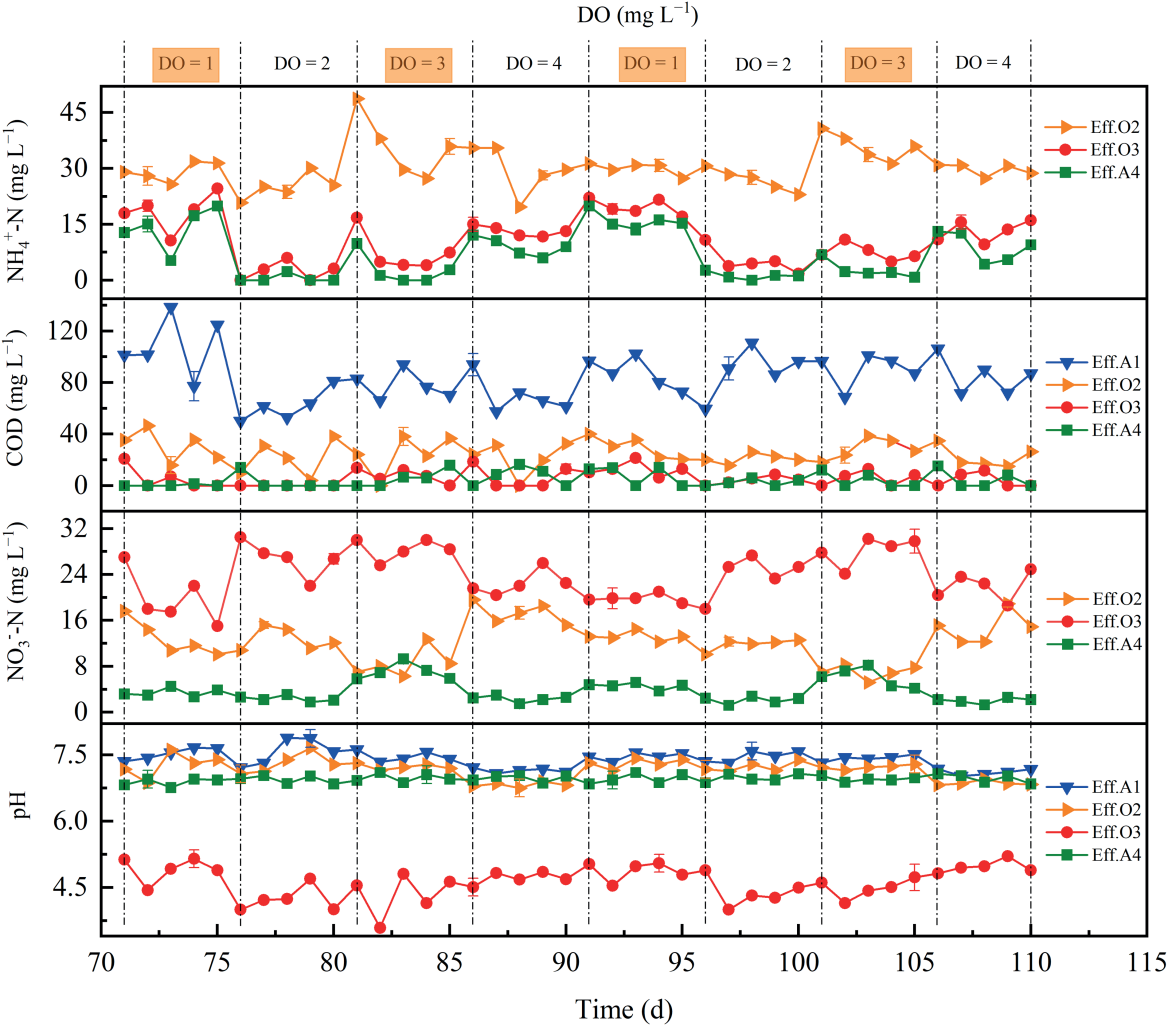
Performance of the reactor during period II.

To verify the adaptability of the reactor under different conditions, the DO concentrations of the chambers O2 and O3 were varied on days 71–110. Continuous aeration was carried out, and the DO concentration was set at 1, 2, 3, and 4 mg L^−1^ successively with repeat batch experiments were adopted, the experimental results are shown in Figure 2. The ARE decreased to 69.9%, resulting in effluent ammonium concentration of reactor to be 15.05 ± 0.62 mg NH_4_^+^-N·L^−1^ for the DO concentration of 1 mg L^−1^. Especially in chamber O3, only a decrease in ammonium concentration of about 10 mg NH_4_^+^-N·L^−1^ was observed, this was worse than the removal performance of ammonium at other DO concentrations (22.2 ± 1.42, 26.4 ± 1.86, and 16.1 ± 1.05 mg NH_4_^+^-N·L^−1^, corresponding to DO concentrations of 2, 3, and 4 mg L^−1^, respectively). The reason for this phenomenon could be attributed to the nitrification activity was weakened when the DO concentrations were lower than 1.3 mg L^−1^ (Kim et al., 2021).

When the DO concentration was set at 2 mg L^−1^, the effluent average ammonium, TNE, and nitrate decreased to 0.83 ± 0.22, 3.55 ± 0.36, and 2.61 ± 0.45 mg L^−1^, respectively, with the TIN removal efficiency in chamber O3 increased from 7.2% (DO = 1 mg L^−1^) to 18.4% (DO = 2 mg L^−1^). As the DO concentration was further increased to 3 mg L^−1^, the average ammonium and nitrate concentrations in the effluent of the reactor were increased to 2.79 ± 0.31 and 5.83 ± 0.27 mg L^−1^, respectively, with the TIN removal efficiency of the reactor decreasing from 92.9% (DO = 2 mg L^−1^) to 82.8% (DO = 3 mg L^−1^). Although the reactor could meet the standard of level A at the DO concentrations of 2 and 3 mg L^−1^ (GB18918-2002, discharge standard of pollutants for municipal WWTPs), while considering that when the DO concentration was 2 mg L^−1^, the reactor achieved better pollutant removal performance and required lower aeration intensity, this may be the optimal DO concentration for the reactor. Unexpectedly, the performance of the reactor was also weakened when the DO concentration was set at 4 mg L^−1^, with the effluent ammonium of the reactor being 9 mg L^−1^, and the nitrate concentration in the O2 effluent was 17.3 ± 1.36 mg L^−1^, which was higher than the nitrate concentration of O2 effluent under other DO concentrations during period II. This may be attributed to the occurrence of heterotrophic denitrification (HD) processes in O2 chamber, since the activity of HD could be inhibited when the DO concentration was higher than 4 mg L^−1^ and the DO is likely to be consumed by heterotrophic bacteria (Gutierrez-Wing et al., 2012; Du et al., 2020), thus leading to the accumulation of nitrate. Moreover, compared with the DO concentrations of 2 and 3 mg L^−1^, a higher ammonium accumulation was observed when the operational DO concentration increased to 4.0 mg L^−1^, the reason may be that the nitrifiers were inhibited and the shape of the biofilm was affected by excessive aeration intensity, which leads to negative effects.

During period II, the COD removal mainly occurred in chambers A1 and O2. A certain amount of COD in O2 could contribute to the occurrence of HD process, while the COD/TIN ratio in O3 was generally lower than 2. Therefore, it was difficult to realize complete HD process in the O3 chamber due to poor COD resources, which could not provide enough electrons (Liang et al., 2021b). Aside from COD measurements, it was found that the change of pH value was closely related to the performance of reactor, when the DO concentration was lower than 4 mg L^−1^, there were no significant differences in the pH values between the inlet and outlet of chamber O2, and decreased only in the effluents of O3 and A4 (Figure 2). It could be speculated that the removal of nitrogen in chamber O2 was probably induced by the simultaneous nitrification and denitrification (SND) process, because one of the features of SND is the acid–base equilibrium before and after the operation (Lai et al., 2020). The occurrence of SND process may benefit from the use of biofilms, which supports the coexistence of anoxic and aerobic conditions (Layer et al., 2020). The decrease in pH and the nitrate accumulation of the O3 effluent were presumably caused by the nitrification process, with the nitrification process being the fundamental mechanism in chamber O3, because the main function of nitrification process is to convert ammonium into nitrate (Pan et al., 2022).When the DO concentration wa s set at 4 mg L^−1^, a decrease in pH of O2 effluent was observed, which was likely attributable to the inhibition of HD caused by the higher DO concentration, thus affecting the acid–base balance of O2 effluent. Notably, when the DO concentrations were 1 and 4 mg L^−1^, a recovery period was likely needed to restore the performance of the reactor, suggesting that the DO concentration is an important factor when the reactor is challenged with the environmental variations.


(4) $$\begin{array}{r} 55S_{0}+50{\mathrm{NO}}_{3}^{-}+38H_{2}O+20{CO}_{2}+4{NH}_{4}^{+}\to \\ 4C_{5}H_{7}O_{2}N+55{\mathrm{SO}}_{4}^{2-}+25N_{2}+64H^{+} \end{array}$$

The pH of the A4 influent was first automatically increased to about 7.0 ± 0.1, and then naturally increased after passing through the oyster shells, with the average pH of the period II further increased to about 7.4 ± 0.1 before the ESAD process. In the reactor, the chamber A4 contributed the most (39.2%–54.6%) to TIN removal efficiency (Supplementary Table S1), and could simultaneously remove ammonium and nitrate in the sewage. However, the accumulation of sulfate is a major problem in the ESAD process. When the DO concentration was 2 and 3 mg L^−1^, the average sulfate productivity was 6.60 ± 0.58 and 6.82 ± 0.48 mg SO_4_^2−^/mg NO_3_^−^-N, respectively, while the sulfate concentration in the effluent of the reactor was always lower than 250 mg L^−1^ (maximum value set by US EPA). According to Eq. 4, the theoretical sulfate productivity was 7.54 mg SO_4_^2−^/mg NO_3_^−^-N, the lower sulfate productivity could be attributed to the use of shell powder in the USS filter, because shell powder contains a small fraction of organic matter, which may facilitate the occurrence of HD process (Liang et al., 2022).

### Performance of the reactor under different COD/TIN ratios

**Figure 3:**
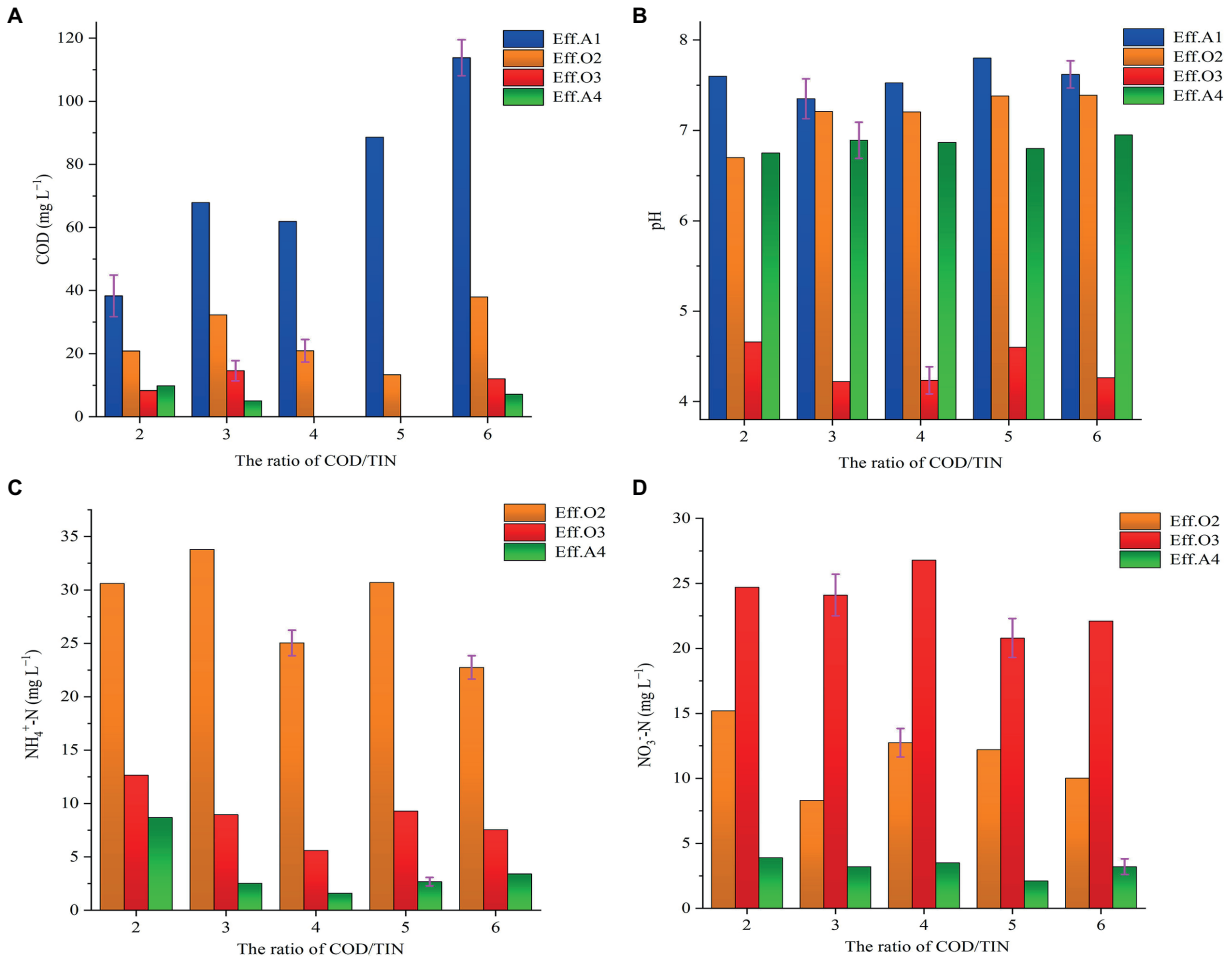
Variations of COD, pH, ammonium, and nitrate in period III of the reactor **(A)** COD; **(B)** pH; **(C)** ammonium; and **(D)** nitrate.

During period III, different COD/TIN ratios were adopted to elucidate the effects on the reactor. Similar to period II, COD was mainly consumed in chambers A1 and O2 during period III (Figure 3). When the COD/TIN ratio was set at 2, the ARE of the reactor was reduced to 82.6% with the ammonium removal effect deteriorated, while this ratio was generally greater than 93% for the COD/TIN ratio of higher than 2. In addition, when the COD/TIN ratio was 2, it was observed that the pH decrease in the O2 effluent was more severe and a higher nitrate concentration was obtained compared to other COD/TIN ratios. This was likely due to the limited COD concentration in the chamber O2, since carbon could act as an energy source for microbes (Xi et al., 2022). Thereafter, with the COD/TIN ratio gradually increased from 2 to 6, the reactor exhibited stable performance and the effluent average concentrations of ammonium and TNE were lower than 5 and 15 mg L^−1^, respectively. Although the COD concentration in the O3 influent was relatively high when the COD/TIN ratio was 6, the nitrate concentration in O3 effluent was not effectively reduced, which proved that lower concentrations of COD had limited effects on denitrification. Overall, the proposed AOOA process demonstrated outstanding performance in removing pollutants from sewage and is also simple to operate.

By comparing phases II and III that ran under the same operating conditions, a higher nitrate concentration was observed in the reactor’s effluent on days 121–125 (8.35 ± 0.58 mg L^−1^), with the MLSS concentration of 17.6 mg L^−1^. This could be explained by the hampering of the mass transfer between the nitrate and the ESAD filter was influenced due to the gradually accumulated sludge in the chamber A4, so it leads to the increase of nitrate in the effluent. To alleviate this potential problem, the oyster shell and ESAD filter were manually taken out and excess sludge was washed away, they were then filled back into chamber A4 successively. For the ESAD filter, only a small amount of sludge was left on its surface for subsequent denitrification process. This resulted in nitrate concentration of 5.23 ± 0.35 mg L^−1^ on days 146–150, suggesting that the removal of excess sludge from ESAD filter could help in improving the denitrification capacity.

### Performance of the reactor to treat real sewage and under vibration conditions

**Figure 4:**
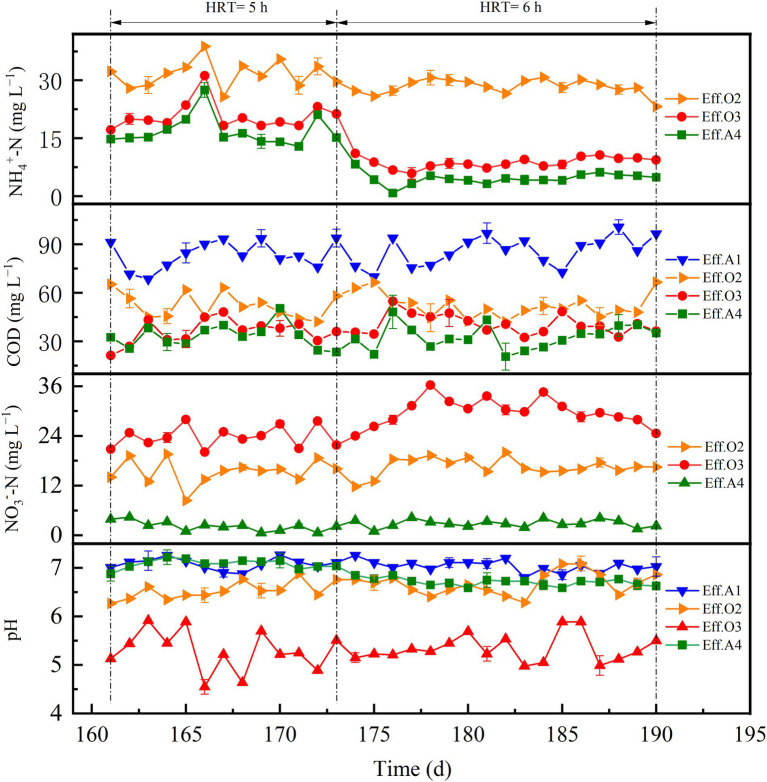
Performance of the reactor during period IV.

Switching from synthetic domestic sewage to real domestic sewage resulted in effluent COD and ammonium concentrations of 32.4 ± 10.3 and 16.8 ± 0.42 mg L^−1^ (days 161–173), respectively, with the ARE decreased to 65.1%. This could have been caused by the activity of the nitrification process, which was suppressed due to the low temperature (Gao et al., 2022), leading to low ARE and a build-up of ammonium in the effluent. Consistent with this study, with the decrease of temperature, other researchers have also found a decrease in the activity of the nitrification reaction (Kim et al., 2006; Hoang et al., 2014). Considering that the reactor could not adapt to the influent load at this time under such cold conditions, the HRT of the reactor was increased from 5 to 6 h to explore whether the risk of effluent ammonium could be reduced. With continuous operation, a recovery trend was observed with the average ARE increased to 90.1% (days 174–190), indicating that at lower temperature, prolonging the HRT is the key method to cope with the decrease of ARE in the reactor. The effluent nitrate was always lower than 5 mg L^−1^ throughout period IV, indicating favourable denitrification performance of the ESAD process, even the average water temperature was only 13°C. Compared with other periods, a higher concentration of effluent COD was observed in period IV (33.6 ± 12.9 mg L^−1^), which may be due to the reason that real domestic sewage contained a certain amount of non-biodegradable organic matter, which led to the increase of remaining COD. Moreover, the average pH value of O2 effluent during period IV decreased to acidic to a certain extent (Figure 4), while the average pH value of O3 effluent was slightly higher than the other periods. This may be due to a decrease in the contribution of HD processes in O2 and an increase in O3.

To control the possible reduction in treatment efficiency of the reactor caused by the gradually thickening biofilms, the vibration method was adopted to remove excess sludge on the biofilms. The overall performance of the reactor on the day of vibrations, the day before the vibrations and the day after the vibrations are recorded in Supplementary Table S2. The results verified that the vibrations had no negative impact on the performance of the reactor. Although the effluent COD of the reactor rose to a certain extent after the vibrations, it was still within the acceptable range (<50 mg L^−1^). Overall, the proposed AOOA process would be an applicable pathway even without the reflux operation, and the implementation of the vibration method also ensures long-term and efficient operation of the reactor.

### Evolution of the microbial community

**Figure 5:**
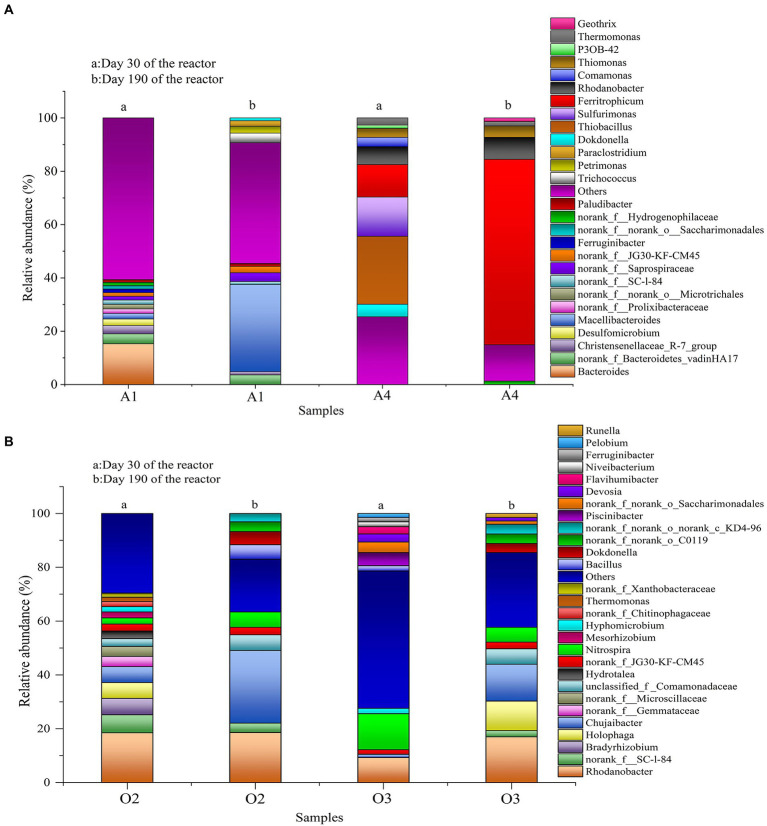
Changes in microbial community in the reactor **(A)** genus level of chambers A1 and A4; **(B)** genus level of chambers O2 and O3.

The samples were collected from the reactor to discover the changes in the microbial communities, distributions of bacterial sequences at phylum and genus levels as shown in Supplementary Table S1 and Figure 5, respectively (relative abundance ≥ 1%). At the phylum level (Supplementary Figure S1), the *Bacteroidetes* was the most dominant phylum in the A1 chamber. Its abundance increased from 35.6% (day 30) to 49.5% (day 190). The enrichment of *Bacteroidetes* could be related to its function, as it has been reported to be mainly used for the degradation of organic matter (Hou et al., 2021). For the chambers O2 and O3, the dominant phyla were *Proteobacteria* and *Bacteroidetes*, their coexistence may be helpful in removing ammonium (Chen et al., 2016). Similar to previous ESAD studies, the phylum *Proteobacteria* was found to be the most abundant bacteria in A4 chamber. This could be due to the fact that *Proteobacteria* can play an important role in degrading the organic matter and nitrate (Zhao et al., 2022).

From the genus assignment results, the *Bacteroides* exhibited a higher proportion than other genera in A1 chamber on day 30 (Figure 5A), this was probably due to its enrichment could promote the degradation of glucose (Wang et al., 2021a). Notably, the *Bacteroides* was not observed on day 190, with greater abundances of *Macellibacteroides*, *Trichococcus*, and *paraclostridium* were observed in A1 chamber, their existences have been confirmed could help to degrade the organic matters (Yan et al., 2020; She et al., 2021; He et al., 2022). The changes in the microbial community within the A1 chamber may be attributed to changes in the influent conditions and environmental factors.

For the chambers O2 and O3, the enrichment of genus *Rhodanobacter* was observed throughout the study (Figure 5B), it is worth noting that the *Rhodanobacter* could be a prominent role in the reactor, because it can degrade the ammonium and convert nitrate into nitrogen gas (Wang et al., 2020). Meanwhile, the genera *Chujaibacter* and *Nitrospira* were also dominant in chambers O2 and O3, the *Nitrospira* is known as a nitrification-related bacterium during nitrification process (Li et al., 2022a), and *Chujaibacter* is reported to be positively correlated with *Nitrospira* (Sato et al., 2021). The presence of genera *Bradyrhizobium*, *Mesorhizobium*, *Dokdonella*, *Ferruginibacter*, *Holophaga*, and *Hyphomicrobium* could be related to the denitrification processes or organic degradation (Chen et al., 2021; Liang et al., 2021b; Wang et al., 2021b; Qian et al., 2022; Li et al., 2022b), this is helpful in removing COD and nitrate in the chambers O2 and O3, and the *unclassified_f __Comamonadaceae* was able to reduce nitrate by using organic carbons (Zhang et al., 2022). In general, the main functions of bacteria in the O2 and O3 chambers may be to get involved in the nitrification process, denitrification process and removal of organic matter, which further verified the removal trend of pollutants in the reactor.

In A4 chamber, the dominant genera were *Thiobacillus*, *Sulfurimonas*, and *Ferritrophicum* on day 30. However, on day 190, the *Thiobacillus* and *Sulfurimonas* were not observed, while the abundance of *Ferritrophicum* increased to 69.6% (Figure 5A). *Thiobacillus* and *Sulfurimonas* were identified as the major denitrifying genera in the ESAD process (Vandekerckhove et al., 2018). Significant changes in their abundances may have been caused by the washing of ESAD filters. Furthermore, *Ferritrophicum* was verified as a denitrification bacterium (Wan et al., 2019), its enrichment is helpful for the denitrification process, which also indicates that although washing ESAD filter will cause changes in the structure of bacterial community, the main bacteria are still the denitrifying bacteria. The existence of *Rhodanobacter* may be that the ESAD filter could block the bacteria in O3 effluent, which helps in realising the ND processes simultaneously, and further contributes to the decrease of sulfate productivity in the effluent.

## Conclusion

The AOOA process was successfully explored to treat domestic sewage. In chambers O2 and O3, the control of DO has a great influence on the removal of pollutants in the reactor. An increase in ammonium concentration was observed in the reactor effluent when the temperature decreased to 13°C ± 2°C, and extending the HRT from 5 to 6 h could alleviate this problem. The AOOA process is a promising method for treating domestic sewage, especially for the domestic sewage which is difficult to be centrally treated, the proposed AOOA process has a simple operation and requires lower investment costs.

## Data availability statement

The raw 16S rRNA sequences have been deposited in the National Center for Biotechnology Information Sequence Read Archive database under accession number PRJNA849697.

## Author contributions

YaW: conceptualization, data curation, investigation, funding acquisition, writing–original draft, and visualization. BL: conceptualization, data curation, formal analysis, and funding acquisition. FK: conceptualization and editing. YoW: funding acquisition. CZ and ZL: supervision. TZ: funding acquisition, writing–original draft, and editing. ZZ: funding acquisition, data curation, writing–original draft, and editing. All authors contributed to the article and approved the submitted version.

## Funding

This work was supported by the National Key Research and Development Program of China (grant no. 2020YFC1806402), Major Key and Core Technology Research Project (direction of water pollution control industry chain), Science and Technology Plan of Shenyang in 2020 (grant no. 20-202-4-37), and the Ningxia Provincial Natural Science Foundation of China (grant no. 2020AAC03272).

## Conflict of interest

The authors declare that the research was conducted in the absence of any commercial or financial relationships that could be construed as a potential conflict of interest.

## Publisher’s note

All claims expressed in this article are solely those of the authors and do not necessarily represent those of their affiliated organizations, or those of the publisher, the editors and the reviewers. Any product that may be evaluated in this article, or claim that may be made by its manufacturer, is not guaranteed or endorsed by the publisher.

## Supplementary material

The Supplementary material for this article can be found online at: https://www.frontiersin.org/articles/10.3389/fmicb. 2022.970548/full#supplementary-material
