# Supplementary material for: An efficient anoxic/aerobic/aerobic/anoxic process for domestic sewage treatment: From feasibility to application
Source: Front Microbiol. 2022 Aug 2;13:970548. doi: 10.3389/fmicb.2022.970548 (PMC9378819; doi:10.3389/fmicb.2022.970548)
Supplement: Supplementary file 1 [file Data_Sheet_1.doc]

Supplementary Material

**FIGURES**


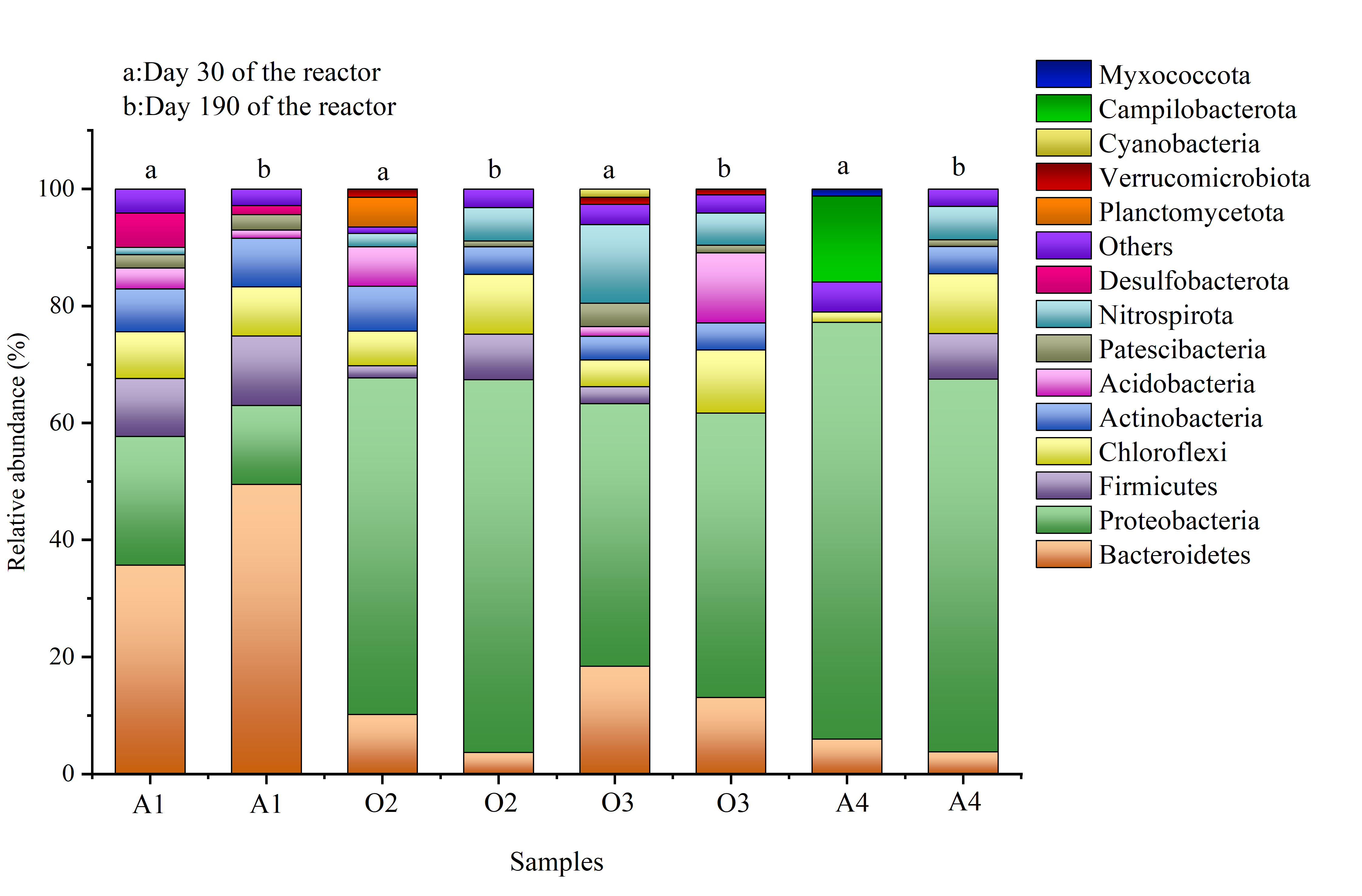


**FIGURE S1** **|** Microbial community of the reactor classified at phylum level.

**TABLE**

**TABLE S1** **|** The TIN removal efficiency of the chamber during period II.

| **DO concentration of O2 and O3 (mg L^-1^)** | | **1** | **2** | **3** | **4** |
| --- | --- | --- | --- | --- | --- |
| The TIN removal efficiency of the chamber (%) | O2 | 14.9 | 23.4 | 11.1 | 6 |
|  | O3 | 7.2 | 18.4 | 17.1 | 22.6 |
|  | A4 | 39.2 | 51.1 | 54.6 | 48 |

**TABLE S2** **|** The effect of vibration on reactor performance.

| **Days** | **119** | **120** | **121** | **139** | **140** | **141** | **159** | **160** | **161** | **179** | **180** | **181** |
| --- | --- | --- | --- | --- | --- | --- | --- | --- | --- | --- | --- | --- |
| Effluent COD of the reactor (mg L^-1^) | 16.3 | 19.3 | 33.6 | 8.3 | 6.8 | 26.6 | 18.9 | 23.6 | 32.6 | 31.5 | 31.1 | 43.3 |
| Effluent ammonium of the reactor (mg L^-1^) | 1.89 | 2.2 | 4.6 | 8.9 | 9.8 | 3.3 | 2.3 | 3.1 | 14.8 | 4.5 | 4.1 | 3.2 |
| TNE of the reactor (mg L^-1^) | 5.6 | 7.3 | 8.3 | 13.8 | 13.9 | 7.6 | 6.8 | 7.7 | 18.8 | 7.5 | 6.5 | 6.7 |
